# Differential Changes in the Lateralized Activity of Identified Projection Neurons of Motor Cortex in Hemiparkinsonian Rats

**DOI:** 10.1523/ENEURO.0110-19.2019

**Published:** 2019-07-08

**Authors:** Alain Rios, Shogo Soma, Junichi Yoshida, Satoshi Nonomura, Masanori Kawabata, Yutaka Sakai, Yoshikazu Isomura

**Affiliations:** 1 Brain Science Institute, Tamagawa University, Tokyo 194-8610, Japan; 2 Graduate School of Brain Sciences, Tamagawa University, Tokyo 194-8610, Japan; 3 Physiology and Cell Biology, Graduate School of Medical and Dental Sciences, Tokyo Medical and Dental University, Tokyo 113-8519, Japan; 4 Department of Anatomy and Neurobiology. University of California, Irvine, Irvine, CA 92697; 5 Dominick P. Purpura Department of Neuroscience, Albert Einstein College of Medicine, Bronx, NY 10461

**Keywords:** continuous activity monitoring, home cage, motion detector, physical activity

## Abstract

In the parkinsonian state, the motor cortex and basal ganglia (BG) undergo dynamic remodeling of movement representation. One such change is the loss of the normal contralateral lateralized activity pattern. The increase in the number of movement-related neurons responding to ipsilateral or bilateral limb movements may cause motor problems, including impaired balance, reduced bimanual coordination, and abnormal mirror movements. However, it remains unknown how individual types of motor cortical neurons organize this reconstruction. To explore the effect of dopamine depletion on lateralized activity in the parkinsonian state, we used a partial hemiparkinsonian model [6-hydroxydopamine (6-OHDA) lesion] in Long–Evans rats performing unilateral movements in a right–left pedal task, while recording from primary (M1) and secondary motor cortex (M2). The lesion decreased contralateral preferred activity in both M1 and M2. In addition, this change differed among identified intratelencephalic (IT) and pyramidal tract (PT) cortical projection neurons, depending on the cortical area. We detected a decrease in lateralized activity only in PT neurons in M1, whereas in M2, this change was observed in IT neurons, with no change in the PT population. Our results suggest a differential effect of dopamine depletion in the lateralized activity of the motor cortex, and suggest possible compensatory changes in the contralateral hemisphere.

## Significance Statement

The motor cortex normally exhibits preferred activation during contralateral, but not ipsilateral, movements. In the parkinsonian state, this contralateral preference may be affected, resulting in impaired bimanual coordination or abnormal mirror movements. However, it remains unknown how individual types of motor cortical neurons are reorganized in the parkinsonian state. Using multiunit recording in hemiparkinsonian rats during unilateral forelimb movements, we observed a reduction in contralateral preference in both primary (M1) and secondary (M2) motor cortices in the lesioned hemisphere. Moreover, using a light-induced spike collision test, we demonstrated that these decreases in lateralized activity were present in PT neurons exclusively in the primary motor cortex, but only in IT neurons in the secondary motor cortex.

## Introduction

Parkinson’s disease (PD) is a neurodegenerative disorder produced by the loss of dopaminergic neurons in the substantia nigra pars compacta (SNc), which leads to an abnormal activity of the cortico-basal ganglia (BG) circuit (Gradinaru et al., 2009). The most striking physical disabilities resulting from these changes include paucity and slowness of movements, stiffness, and tremor (Galvan and Wichmann, 2008). Because the locus of primary pathology is in the SNc, PD research has focused on the BG. However, dopamine depletion in one hemisphere may not only disturb the function of the BG, but also affects frontal areas of the cortex in the same side and produces compensatory shifts in activation toward motor and non-motor areas of the contralateral hemisphere (Galvan and Wichmann, 2008; Pasquereau and Turner, 2011; Shepherd, 2013; Pasquereau et al., 2016).

Neuroimaging and electrophysiological studies in humans and monkeys have shown that unilateral movements are associated with preferred activation (lateralization) of the contralateral primary motor cortex (M1), premotor cortex (PM) and supplementary motor area (SMA; Colebatch et al., 1991; Catalan et al., 1998; Mattay et al., 1998; Solodkin et al., 2001; Kurata, 2007). However, there is a difference in the degree of lateralization among M1 and other motor areas: the preferred activation during contralateral movements in M1 contrasts with the bilateral activation of PM (Kurata, 2007; Soma et al., 2017, 2019). Imaging studies have revealed that the lateralization in the motor cortex is diminished in PD (Wu et al., 2015). Moreover, parkinsonian patients and animal models exhibit reduced activity in the contralateral BG or motor thalamic nuclei (Donoghue and Wise, 1982; Taha et al., 1996; Boraud et al., 2000; Levy et al., 2001; Pessiglione et al., 2005; Baker et al., 2010). Patients with PD commonly exhibit motor symptoms related to abnormal selective limb representation, such as impaired bimanual coordination or mirror movements (i.e., simultaneous contralateral, involuntary, identical movements that accompany voluntary movements; Espay et al., 2005), circumstances that have been attributed to the loss of this lateralized activity pattern (Johnson et al., 1998; van den Berg et al., 2000; Almeida et al., 2002; Vidal et al., 2003; Espay et al., 2005; Li et al., 2007; Ottaviani et al., 2008; Borgheresi et al., 2010; Wu et al., 2010, 2015).

Rodents are suitable for investigations of motor information processing due to the possibility of genetic and optical manipulation and identification of specific neurons and circuits in these animals. Also, the use of head-fixed rodents has been useful for the precise study of forelimb movements in a stable posture (Kimura et al., 2012; Saiki et al., 2018). By contrast, most hemiparkinsonian rat studies used anesthetized or freely moving animals (Magill et al., 2001; Mallet et al., 2008; Lemaire et al., 2012; Delaville et al., 2015).

In rodents, M1 and M2 are homologous to primate M1 and PM/SMA, respectively (Donoghue and Wise, 1982; Neafsey et al., 1986; Reep et al., 1987; Rouiller et al., 1993; Deffeyes et al., 2015; Hira et al., 2015). Previous studies show that M1 neurons undergo greater activation during contralateral movements than M2 neurons, supporting the idea of functional diversity between rodent motor cortices (Soma et al., 2017, 2019). In addition, the motor cortical areas have two classes of projection neurons with specific morphologies and axonal projections: intratelencephalic (IT) and pyramidal tract (PT) neurons (Shepherd, 2013, 2014). IT neurons send projections bilaterally to other areas within the telencephalon. By contrast, PT neurons send projections to the ipsilateral cortex, striatum, thalamus, pontine nuclei, and contralateral spinal cord, but do not send bilateral telencephalic projections (Reiner et al., 2003; Morishima and Kawaguchi, 2006). In accordance with the anatomic traits of these neurons (Shepherd, 2014; Harris and Shepherd, 2015), PT neurons represent contralateral movements more preferentially than IT neurons in M1 and M2 of primates (Tanji et al., 1987) and rodents (Soma et al., 2017). The parkinsonian state produces specific changes in these cortical projection neurons (Shepherd, 2013). Studies of identified projection neurons in M1 in hemiparkinsonian primates revealed selective abnormalities in the PT population, including reduced resting firing rates, an elevated tendency to fire action potentials in irregular patterns, and a weakened encoding of movement kinematics (Pasquereau and Turner, 2011; Pasquereau et al., 2016). However, we have yet to elucidate the control of ipsilateral and contralateral movements in parkinsonism, as well as how specific neuronal populations are reorganized under this condition.

In this study, we analyzed the effect of partial unilateral striatal dopamine loss in the lateralized activity of M1 and M2, and possible compensatory changes in the contralateral hemisphere. For this purpose, we used an optogenetic spike collision method in transgenic rats to identify two kinds of projection neurons (IT and PT) and interneurons. During the recording sessions, the rats performed a two-pedal behavioral task that required unilateral movements. In a partial lesioned hemiparkinsonian model, the rats were able to successfully perform the task with both forelimbs, but still exhibited some of the characteristic parkinsonian behaviors and changes in cortical activity (Sauer and Oertel, 1994; Brazhnik et al., 2012; Hernandez et al., 2013; Guo et al., 2015; Jávor-Duray et al., 2015).

## Materials and Methods

### Animals and surgery

All experiments were approved by the Animal Research Ethics Committee of Tamagawa University and were performed in accordance with the Fundamental Guidelines for Proper Conduct of Animal Experiment and Related Activities in Academic Research Institutions (Ministry of Education, Culture, Sports, Science, and Technology of Japan) and the Guidelines for Animal Experimentation in Neuroscience (Japan Neuroscience Society). All surgical procedures were performed under appropriate isoflurane anesthesia (see next paragraph and 6-OHDA lesion), and all reasonable efforts were made to minimize suffering.

Eighteen adult W-TChR2V4 Long–Evans rats (253 ± 41 g, males) that expressed the ChR2-Venus conjugate under the control of the Thy1.2 promoter (Tomita et al., 2009) were kept in their home cage under an inverted light schedule (lights off at 9 A.M., lights on at 9 P.M.). The rats were briefly handled by the experimenter (10 min/d on 2 d) before the surgery. For implantation of the head-plate (CFR-2, Narishige) and injection of 6-hydroxydopamine (6-OHDA), animals were anesthetized with isoflurane (4.5% for induction and 2.0–2.5% for maintenance; Pfizer) using an inhalation anesthesia apparatus (Univentor 400 anesthesia unit, Univentor) and placed on a stereotaxic frame (SR-10R-HT, Narishige). For local anesthesia, lidocaine (AstraZeneca) was administered around the surgical incisions. Reference and ground electrodes (Teflon-coated silver wires, A-M Systems; 125 μm in diameter) were implanted above the cerebellum. During anesthesia, body temperature was maintained at 37°C using an animal warmer (BWT-100, Bio Research Center). Analgesics and antibiotics were applied postoperatively as required (1 mg/kg meloxicam s.c.; Boehringer Ingelheim; gentamicin ointment, 0.1%, MSD). In some experiments [seven hemiparkinsonian (13 sessions) and six healthy rats (six sessions)], a twisted Teflon-coated silver wire electrode was implanted into both upper forelimbs (near the biceps brachii muscle) for simultaneous monitoring of EMG activity in both forelimbs. After full recovery from surgery (5 d later), rats had *ad libitum* access to water for 1 d/week, but during the rest of the week could obtain water only by performing the task correctly. When necessary, an agar block (containing 15 ml of water) was given to the rats in their home cage to maintain them at 85% of original body weight (Saiki et al., 2014).

### 6-OHDA lesion

6-OHDA injections were performed in the same surgical session as for the head-plate implant. Two doses of 3 μl of solution containing 12 μg of 6-OHDA bromide (Sigma) in 0.9% saline NaCl stabilized with 0.05% ascorbic acid, were injected trough a glass capillary with a 50-μm tip into two right dorsolateral striatum (DLS) locations with the following coordinates: AP +1, ML +3.5, DV –4 mm, and AP +0.2, ML +3.8, DV –4.5 mm. A similar amount of 0.9% saline NaCl was injected in the left hemisphere (Lemaire et al., 2012; Hernandez et al., 2013; Guo et al., 2015). Injections were administered at 0.1 μl/min using an automatic injection pump. After the injection was completed, the capillary was left in place for five additional minutes to allow diffusion and to prevent the solution from flowing back up the guide. Desipramine hydrochloride solution (25 mg/kg, Sigma) was injected intraperitoneally 30 min before 6-OHDA injection to protect noradrenergic neurons (Lemaire et al., 2012). The dopamine neuron lesion was assessed 14 d after 6-OHDA injection by challenge with apomorphine (0.05 mg/kg, s.c.; Schwarting and Huston, 1996). Those animals that made ≥150 net contraversive rotations in 1 h were considered for recording sessions.

### Behavioral task

**Figure 1.**
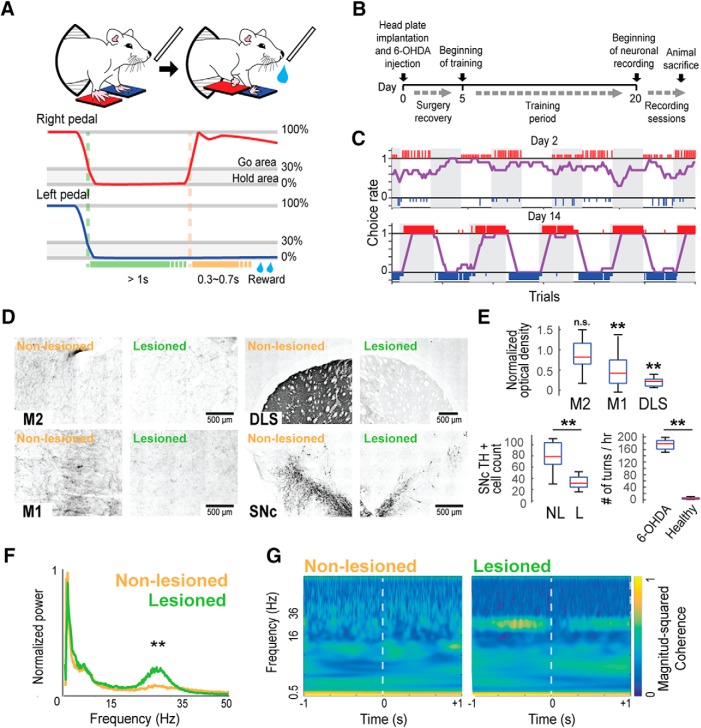
Behavioral task in hemiparkinsonian rats. ***A***, Schematic of the right–left pedal task (top) and a representative of the pedal trajectory during one correct trial in the right-rewarded block (middle and bottom). Rats push both pedals for at least 1 s and release only one limb (depending on which movement is being rewarded in that block) to receive a reward. ***B***, Experiment design timeline. ***C***, Representatives of task performance at day 2 (top) and day 14 (bottom). Large and small colored vertical bars (red, right choice; blue, left choice) indicate correct and incorrect trials, respectively. The choice rate of the right pedal (purple line) was calculated by averaging the number of right choices obtained from the past 10 trials. ***D***, Representative images of M2, M1, DLS, and SNc showing TH immunostaining difference between the non-lesioned and lesioned hemisphere. ***E***, TH staining optical density values of the lesioned hemisphere normalized to the non-lesioned hemisphere in M2, M1, and DLS (top); number of SNc TH+ cells in the non-lesioned (NL) and lesioned (L) hemisphere (bottom left); difference of total turns in 1 h after apomorphine injection between healthy and hemiparkinsonian rats (bottom right); **p < 0.01, n.s.: not significant, unpaired *t* test. ***F***, Population averaged M1 LFP power spectra around the onset of contralateral pedal release (±1.5 s) showing the LFP power and peak frequency in the β range (15–35 Hz) in the lesioned hemisphere; **p < 0.01, Wilcoxon rank-sum test. ***G***, Wavelet coherence between M1 and M2 LFP signals around the onset of contralateral pedal release. Coherence was normalized to fall between 0 and 1.

We used a right–left pedal task in a custom-made operant conditioning system (custom-made by O’Hara & Co., Ltd.) to examine the selectivity of contralateral or ipsilateral forelimb movement in the neuronal activity of the motor cortices (M1 and M2) in rats (Fig. 1*A*, top). In this task, the rats had to manipulate the right and left pedals with the corresponding forelimb in a head-fixed condition. They spontaneously started each trial by pushing both pedals down and holding them for a short period (“holding period,” at least 1 s; within a holding area, 0–30% in relative pedal position). After the holding period was completed, they had to choose either the right or left pedal without any instruction cue, and then release it to obtain 0.1% saccharin water (10 μl) as a reward (Fig. 1*A*, bottom). This task consisted of two blocks, right pedal–rewarded and left pedal–rewarded, in which the rats had to choose the appropriate pedal depending on the context. Each block lasted until the rat performed at least 30 correct trials and achieved a correct performance of 80% in the last 10 trials. The reward was dispensed from the tip of a spout by a micropump with a 300- to 700-ms delay (100-ms steps at random). The reward delivery period was followed by a short intertrial interval (1 s). If the rats chose the incorrect pedal, they were not rewarded (error trial), and only an error sound was given (3 kHz, 300 ms). If they did not complete the holding period (immature trial), no feedback was given (no reward and no error sound). The rats typically learned this task within two weeks (training for 2–3 h/d; Fig. 1*B*,*C*).

Once the rats completed the operant learning of the right-left pedal task, they underwent a second surgery under isoflurane anesthesia, and tiny holes (1.0–1.5 mm in diameter) were made in the skull and dura mater above M1 (1.0 mm anterior, 2.5 mm lateral from bregma) and M2 (3.5 mm anterior, ± 2.4 mm lateral from bregma; Saiki et al., 2018; Yoshida et al., 2018). These coordinates were determined by intracortical microstimulation (50–100 μA, 50 pulses at 100 Hz) to evoke reliable movements of the contralateral forelimb in preliminary experiments (data not shown). In addition, after the recordings, the coordinates were confirmed to evoke reliable movements of the contralateral forelimb by optogenetic stimulation with blue light-emitting diode (LED) light pulses (460 nm, 5–10 mW, 20 pulses at 100 Hz). To perform the optogenetic light-induced spike collision in a subset of rats (*n* = 13; see Materials and Methods, Optical (optogenetic) stimulation), additional tiny holes were made above the pontine nuclei (7.0 mm posterior, 1.0 mm lateral from bregma). All holes were immediately covered with silicone sealant (DentSilicone-V, Shofu).

### Electrophysiological recording

We performed extracellular multineuronal (multiple isolated single-unit) recordings from individual neurons in the output layer of motor cortices while the rats performed the behavioral task. All recording sessions were performed after the two weeks of training period, once the rats’ performance reached a plateau (Fig. 1*B*). For this purpose, 32-channel silicon probes (ISO-3x-tet-A32; NeuroNexus Technologies) supported by agarose gel (2% agarose-HGT, Nacalai Tesque) were precisely inserted into M1 and M2 at depths of up to 1250 μm deep (putative layer 5). Insertions were performed using fine micromanipulators (SM-15 and SMM-200B, Narishige) at least 1 h before the start of the recording experiment. The wide-band signals were amplified, filtered (FA64I, Multi-Channel Systems; final gain, 2000; bandpass filter, 0.5 Hz to 10 kHz) through a 32-channel head-stage (MPA32I, Multi-Channel Systems; gain, 10). The signals were digitized at 20 kHz and recorded by three 32-channel hard-disk recorders (LX-120, TEAC), which simultaneously digitized the pedal positions tracked by angle encoders and the optogenetic stimulation events. In some rats (*n* = 7), the biceps EMG activity of both forelimbs was obtained by an amplifier with a head-stage (EX4-400, Dagan; gain, 1000; bandpass filter, 0.3 Hz to 10 kHz). This signal was also digitized at 20 kHz and recorded using the 32-channel hard-disk recorder.

### Optical (optogenetic) stimulation

In some sessions, we applied the Multi-Linc (multiareal/multineuronal light-induced collision) method, which enabled us to effectively identify pyramidal neurons sending direct projections to specific areas by combining multiareal optogenetic stimulation and multineuronal recordings. Before insertion of the silicon probes, an optical fiber (FT400EMT, FC, Thorlabs; NA, 0.39; internal/external diameters, 400/425 μm) for stimulation was placed on the surface of either M1 or M2, and another optical fiber was vertically inserted into the pontine nuclei (9000 μm deep) using micromanipulators (SM-25A, Narishige). To evoke antidromic spikes in specific axonal projections from the IT or PT neurons of M1 or M2 (IT neurons: contralateral M1 or M2; PT neurons: ipsilateral pontine nuclei), a blue LED light pulse (intensity, 5–10 mW; duration, 0.5–2 ms, typically 1 ms) was applied through each of the two optical fibers using an ultra-high-power LED light source (UHP-Mic-LED-460, FC, Prizmatix) and a stimulator (SEN-8203, Nihon Kohden). To be classified as projecting neurons, neurons were required to meet several criteria, including constant latency, fixed frequency (frequency-following test, two pulses at 100 and 200 Hz), and collision test (Lipski, 1981; Soma et al., 2017; Saiki et al., 2018). The online collision test was, at the time, just tentative to readily accumulate spike collision data that would be sufficient for *post hoc* analysis, completing multineuronal collision tests (Saiki et al., 2018).

### EMG data analysis

**Figure 2.**
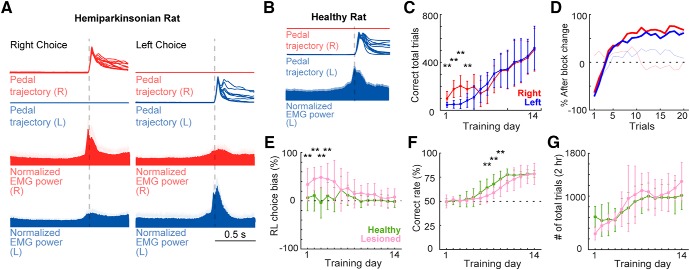
Task performance. ***A***, top, Pedal trajectories of right and left forelimbs during task performance in hemiparkinsonian rat. Middle and bottom, Averaged population EMG activity (±SEM) of both forelimbs aligned to pedal-release onset, during right or left forelimb movements (left and right columns, respectively). ***B***, EMG activity of left forelimb in healthy rat showing left muscles activity change associated with movement execution of the left forelimb. ***C***, Rate of correct responses for each forelimb in hemiparkinsonian rat. In the first 4 d of training, the number of correct trials was higher for the right forelimb than the left forelimb; **p < 0.01, two-way ANOVA with Tukey–Kramer *post hoc* test. ***D***, Correct rate after block change in hemiparkinsonian rat. Solid lines represent the number of trials needed to correctly change the response after a block change in training day 14. At the end of the training period, this number was similar for both forelimbs. The faint lines represent training day 6. ***E***, The rat chooses the correct pedal based on the reward. Relative to healthy rats, lesioned rats exhibited a higher bias toward the right in the initial 4 d of training; **p < 0.01 compared to the corresponding day of healthy group, two-way ANOVA, Tukey–Kramer *post hoc* test. ***F***, Rate of correct responses. The healthy rats showed a higher rate of correct responses during days 7–9, choosing the rewarded pedal >75% of the time; **p < 0.01 compared to the corresponding day of healthy group, two-way ANOVA with Tukey–Kramer *post hoc* test. ***G***, Number of total trials per session.

The EMG signal was rectified to calculate the onset time of muscle activity. The onset time was determined by the first of 10 consecutive 1-ms bins (10 ms) in which the EMG power deviated by 5 SD of the mean value calculated during the baseline period (–1000 to –700 ms relative to the onset of pedal release; Fig. 2*A*). We determined EMG peak activity as the mean of the normalized activity in a time window of ±150 ms around the maximum EMG activity associated with pedal movement.

### LFP data analysis

For every correct trial, we acquired the M1 local field potential (LFP) data of a window of ± 1 s around pedal release. We filtered the LFP signals with a bandpass filter (0.5–100 Hz), and rectified them (Mukovski et al., 2007). In the first step, the power spectra of LFP signals were calculated; here, power measures the intensity of a signal as a function of its frequency. Data were transformed from time domain to the frequency domain by fast Fourier transformation (Brillinger, 1983), at a sampling rate of 1000 Hz. Next, we calculated wavelet coherence between LFP of M1 and M2, using the MATLAB function *wcoherence* (MathWorks), which measures the correlation between two signals in the time–frequency plane. Coherence ranged between 0 and 1, with 1 representing an ideal linear relationship between the signals, and 0 no linear relationship.

### Spike isolation

Raw signal data were processed offline to isolate spike events of individual neurons in each tetrode of the silicon probes. Briefly, spike candidates were detected and clustered using the semiautomatic spike-sorting software, EToS (http://etos.sourceforge.net/; Takekawa et al., 2010, 2012). The spike clusters were further combined, divided, and/or discarded manually to refine single-neuron clusters, based on the presence or absence of refractory periods (2 ms) in the auto-correlograms and cross-correlograms with other clusters, using the manual clustering software Klusters and the viewing software NeuroScope (Hazan et al., 2006).

### Spike collision analysis

To identify IT and PT neurons, we used the Multi-Linc method with *post hoc* analysis to perform multineuronal collision tests (Soma et al., 2017; Saiki et al., 2018). After offline sorting for spike isolation, we compared filtered tetrode (four-channel) traces that had no spikes before the stimulus with those that had a spike in one cluster; these analyses were performed using MATLAB. If we found antidromic-like (all-or-none and no-jittering) spike activities with short latency in many of the control traces, we set a time window for counting possible antidromic spikes, based on clear dissociation between averaged control and test traces due to the presence or absence of spikes. The cutoff threshold, defined in a receiver operating characteristic curve for the distribution of most negative points within the time window, was used to determine whether the spikes were present; thus, we obtained spike and no-spike counts for the control and test events. According to this method, we included spike clusters with a control spike probability of 50% and a test spike probability less than half that of the control. Finally, the passing of the collision test was statistically justified by a 2 × 2 χ^2^ test (*p <* 0.05) for spike and no-spike counts in control and test events (Saiki et al., 2018). The latency of antidromic spikes was defined as the time from the onset of stimulation to the median (the second quartile, 50%) of their peak positions within the time window, and their jitter was defined as the time between the first (25%) and third (75%) quartiles of their peak positions within the time window. In this manner, we judged these spikes to be antidromic or not based on the collisional disappearance of antidromic spikes (collision test), as well as their all-or-none properties, absence of jitters (constant latency test: 0.5 ms), and high reliability (frequency-following test, if applicable in the tentative collision test).

### Analysis of neuronal activity

In each neuron (spike cluster), basal spiking properties and functional activity in relation to behavioral task performance were analyzed using MATLAB, as follows. All spikes occurring for 1 s after optical stimulation were excluded from spike data for subsequent analyses. Ongoing activity was defined as the average firing rate excluding the activity during a time window of –1 to +0.5 s around pedal movement. Spike duration was the time from spike onset to the first positive peak, and the spike width refers to the elapsed time above the half-amplitude of the positive spike wave form. Peak activity was the average firing rate during fifteen 20-ms bins centered at the maximum spike rate. Spike clusters other than identified IT and PT neurons were classified as regular-spiking (RS) and fast-spiking (FS) neurons based on spike duration (>0.6 ms for RS neurons; Isomura et al., 2009; Saiki et al., 2018). As expected, all IT and PT neurons satisfied the criteria for RS neurons (IT neurons: 1.01 ± 0.13 ms, 0.7–1.25 ms, PT neurons: 1.04 ± 0.15 ms, 0.7–1.25 ms). We compared the lesioned hemisphere with the vehicle injected hemisphere in the same hemiparkinsonian rat, according to previous studies (Lemaire et al., 2012; Hernandez et al., 2013). Additionally, for comparison, we used previously acquired data from 16 healthy Long–Evans male rats (Soma et al., 2017). Because we had several groups of neurons (RS vs FS, IT vs PT, and M1 vs M2) under different conditions [healthy (H), non-lesioned side (NL), and 6-OHDA-lesioned side (L) in hemiparkinsonian rats], for simplicity, we use abbreviated expressions to refer to each group, for example, we refer to the RS neurons in M1 in the lesioned hemisphere as M1-RS-L.

Next, we examined functional (task-related) spike activity in relation to the behavioral performance of forelimb movements. To interpret the relationship between functional activity and right–left forelimb control, we primarily focused on neuronal activity recorded during unilateral (contralateral and ipsilateral) forelimb movements (excluding bilateral movements). Thus, spike trains obtained only from unilateral forelimb movement trials were aligned with the onset (0 ms) of pedal release (following the 1-s holding time) during task performance (≧20 trials with total ≧250 spikes). A pedal-release event was detected when the pedal was released outside of the holding area (30% in relative pedal position), and the onset of pedal release was defined as the nearest time point at which the pedal position exceeded 5% of the value before the pedal release event. To define “task-related” activity, the cumulative distribution of all spike positions in the time course of each trial was compared with the distribution of the same number of uniformly distributed spike positions using the Kolmogorov–Smirnov (KS) test, using the *p* value as the task relevance index, where *p* < 10^−6^ was judged as task-related under our analytical conditions (Saiki et al., 2014; Kimura et al., 2017; Soma et al., 2017). A neuron was defined as task-related if it had a smaller task relevance index (*p* < 10^−6^) in either a contralateral or ipsilateral trial; task-related neurons were further classified into three categories on the basis of the task relevance index: contralateral (contralateral, *p* < 10^−6^; ipsilateral, *p* > 10^−6^), ipsilateral (contralateral, *p* > 10^−6^; ipsilateral, *p* < 10^−6^), and bilateral (contralateral, *p* < 10^−6^; ipsilateral, p < 10^−6^).

Furthermore, we classified task-related neurons as Go-type and Hold-type based on their preferred activities (either contralateral or ipsilateral trials) with the smaller task relevance indices. First, we classified neurons that exhibited their peak-activity timings in the peri-event time histograms (PETHs; 20-ms bins) after onset of pedal release as post-movement neurons. Neurons exhibiting their peak-activity timings before pedal-release onset were further divided into two groups according to the dependence of premovement activity on holding time (Soma et al., 2017, 2019). We measured each time point crossing the 75% level of the peak spike activity in all trials for each of the PETHs obtained from different holding time trials (1.0–1.5, 1.5–2.0, 2.0–2.5, and 2.5–3.0 s) and estimated the slope from the plot of cross points versus holding times. Neurons with slopes <0.5 were classified as hold-related neurons (Hold-type), and those with slopes >0.5 were grouped together with the post-movements neurons. These latter two groups exhibited a uniform peak activity distribution and were classified as Go-type neurons.

To evaluate the degree of lateralized activity for each neuron, we compared the peak activities (peak firing rate during –1000 to +500 ms relative to the onset of pedal release) for Go-type, between contralateral and ipsilateral trials. In addition, we confirmed the laterality based on normalized peak activities for Go-type neurons, as follows:

$$Laterality\;index=\{\begin{array}{c} (c-i)/(c+i),if\;c>0\;and\;i>0\\ +1,if\;c>0\;and\;i<0\\ -1,if\;c<0\;and\;i>0 \end{array}$$

where *c* and *i* are the activities associated with contralateral and ipsilateral movements, respectively (Soma et al., 2017, 2019). These parameters were obtained from the following equation:

$$c,i=FR_{peak}/FR_{baseline}-1$$

where *FR_peak_* is the mean firing rate of the peak period (peak ± 150 ms), and *FR_baseline_* is the mean firing rate during the baseline period (–1000 to –700 ms relative to the onset of pedal release). The laterality index ranged from –1 to +1, indicating ipsilateral and contralateral movement-related activity, respectively.

### Histologic observations

After recording experiments were completed, animals were deeply anesthetized with urethane (2–3 g/kg, i.p.; Nacalai Tesque) and perfused transcardially with cold saline followed by 4% formaldehyde in 0.1 mol/L phosphate buffer. Whole brains were postfixed and sliced coronally into 50-μm serial sections using a microslicer (VT1000S, Leica). The sections were Nissl-stained with Neutral Red (Nacalai Tesque) or performed immunostaining for tyrosine hydroxylase (TH). Optical fiber, electrode tracks and TH staining were observed in the motor cortices, DLS, SNc, and pontine nuclei under a microscope (BX51N or IX83P2ZF, Olympus).

### TH staining

After washing in PBS, sections were preincubated in blocking serum (5% normal swine serum in PBS) for 60 min, and then incubated with rabbit anti-rat TH (Abcam) diluted 1:400 in PBS + 0.3% Triton X-100. Between incubation steps, washes were performed in PBS. After incubation with biotinylated donkey anti-rabbit IgG (Abcam; 1:100), peroxidase was visualized using the ABC Immunoperoxidase kit (Vector Laboratories; 1:200) with 3,3’- diaminobenzidine tetrahydrochloride dihydrate (Sigma; 0.5 mg/ml) as the chromogen. Contrast was intensified with 1% nickel ammonium sulfate (Nacalai Tesque). Sections were dehydrated, cleared, and mounted on slides with Entellan(R) new (MERCK) for microscopy. Quantification of TH was performed in 6 rats using ImageJ 1.51 s by calculating optical density (Nonomura et al., 2018), using four continuous sections spanning each of the next areas: M1(AP +1 mm), M2 (AP +3.5 mm), DLS (AP +0.2 mm). All values of axon density were normalized against that of the non-lesioned hemisphere and averaged for each hemisphere. In addition, we quantified the TH+ cells in the SNc using four separate sections through the mesencephalon for each rat, between 5.0 and 5.5 mm posterior from bregma (Metz et al., 2004). We used the medial terminal nucleus to differentiate between the SNc and the VTA. Where this was not possible, SNc boundaries were established according to the stereotaxic atlas of Paxinos and Watson (Paxinos and Watson, 2007). TH+ SNc cell counts from each of the four sections for each rat, performed manually with the aid of the cell counter plugin ImageJ, were separately averaged for each hemisphere and used for statistical analysis. TH+ neurons were identified and defined as densely stained cell bodies visible on the sections.

### Experimental design and statistical analyses

In this study, we used 18 adult male W-TChR2V4 Long–Evans rats to investigate the effect of a 6-OHDA lesion on the selectivity for contra- or ipsilateral forelimb movement representations of M1 and M2 neurons. For comparison, we also used previous recordings from healthy rats (16 adult W-TChR2V4 Long–Evans male rats; Soma et al., 2017). W-TChR2V4 rats express ChR2-Venus in neurons ubiquitously throughout the brain (Saiki et al., 2018), which is useful for optogenetically evoked spike collision tests to identify IT and PT neurons, as described above. For the neuronal analysis, we compared neuronal activity from healthy rats and hemiparkinsonian rats under task performance: healthy rats (834 M1 neurons and 1316 M2 neurons), non-lesioned hemisphere in hemiparkinsonian rats (3174 M1 neurons and 3144 M2 neurons), and lesioned hemisphere in hemiparkinsonian rats (3578 M1 neurons and 3771 M2 neurons). These neurons were further divided into subclasses: RS and FS neurons, task-related and non-task-related neurons, IT and PT neurons. They were compared using appropriate statistical tests (i.e., unpaired *t* test, Mann–Whitney test, Wilcoxon signed-rank test, χ^2^ tests, KS test, Kruskal–Wallis with Dunn’s *post hoc* test, and ANOVA with Tukey–Kramer *post hoc* test). These statistical tests were conducted using MATLAB’s Statistics and Machine Learning Toolbox. Differences were considered statistically significant when *p* < 0.05 (see Results). Unless otherwise noted, data in the text and figures are given as means ± SD (unless otherwise noted) and sample number (*n*).

## Results

### 6-OHDA partial lesion

To develop a hemiparkinsonian rat model suited for the study of motor cortex activity during contralateral and ipsilateral forelimb movements, we partially lesioned the dopaminergic system injecting 6-OHDA directly into the right DLS. This lesion was evident as a decreased of TH staining around the injection area corresponding to the DLS. We quantified the degree of dopamine depletion comparing the TH staining optical density between the lesioned and non-lesioned hemisphere in M1, M2 and DLS, and the number of TH+ cells in the SNc (Fig. 1*D*). The optical density in M2 was similar between the lesioned and the non-lesioned hemisphere (*t* = 1.56, *p* = 0.2, unpaired *t* test). In M1, we found a decrease in the optical density (47% relative to the non-lesioned hemisphere; *t* = 4.27, *p* = 2.7 × 10^−3^, unpaired *t* test). The highest reduction in the TH optical density was observed in the DLS, around the injection site (20% relative to the non-lesioned hemisphere; *t* = 13.82, *p* = 2.6 × 10^−8^, unpaired *t* test; Fig. 1*E*, top). Also, we observed an important decrease of the number of TH+ cells in the SNc (non-lesioned: 79.6 ± 26 cells; lesioned: 34.68 ± 11.34 cells; *t* = 6.01, *p* = 2.37 × 10^−5^, unpaired *t* test; Fig. 1*E*, bottom left). Thus, even with a local DLS 6-OHDA injection, the dopamine depletion was not limited to the injection side, but was also found in M1, along with an important loss of TH+ cells in the SNc. The rotational behavior induced by subcutaneous injection of 0.05 m/kg apomorphine was evident, with at least 150 contralateral turns in 1 h in all recorded rats (*n* = 18), higher than in healthy rats (*n* = 5; *t* = 19.75, *p* = 4.8 × 10^−15^, unpaired *t* test; Fig. 1*E*, bottom right). Furthermore, M1 LFP recordings in the lesioned hemisphere exhibited two characteristic changes of the parkinsonian state: an increase in β-band power relative to the non-lesioned hemisphere [total power (mV^2^ × 10^−3^) 15–35 Hz; non-lesioned: 0.54 ± 0.2, lesioned: 1.02 ± 0.2; *z* = 4.12, *p* = 3.7 × 10^−5^, Wilcoxon signed-rank test; Fig. 1*F*], and an increase in the coherence of the β-frequency between M1 and M2 (Fig. 1*G*; Silberstein et al., 2005; Degos et al., 2009). These results confirm that the consequences of the partial dopaminergic lesion resembled the behavioral and electrophysiological changes associated with the parkinsonian state, but were milder.

### Behavioral changes produced by the partial unilateral 6-OHDA lesion

We used the right–left pedal task to examine the laterality of forelimb movement representation in motor cortex (Fig. 1*A*). In this task, the holding area corresponded to the 30% of the total pedal movement range. Even if rats moved their forelimbs within the holding area, this time was also included in the holding period. These subtle movements during the holding period were similar in both forelimbs, and were much smaller than the movement required to pass out the holding area (right, 0.9 ± 1.2%; left, 1.0 ± 1.1%), indicating that the rats stably pushed both pedals during the holding period. In some rats (*n* = 7, 18 sessions), we recorded the EMG activity of both upper forelimbs simultaneously during the recording sessions. There was no significant difference between the peak right and left forelimb EMG activity during the corresponding forelimb movements (change in EMG between pre- and post-movements: right choice/right-EMG: 45.4 ± 15.4%, left choice/left-EMG: 56.4 ± 17.2%, *z* = 1.02, *p* = 0.3, Wilcoxon rank-sum test; Fig. 2*A*). The slight muscle activity of the opposite forelimb was not associated with a significant pedal movement, and was similar during right and left unilateral movements. This was confirmed in all rats examined (change in EMG between pre- and post-movements: left choice/right-EMG: 10.3 ± 3.8%; right choice/left-EMG: 8.4 ± 3.1%; *z* = 0.56, *p* = 0.5, Wilcoxon rank-sum test). The mean onset time of forelimb movement, relative to the onset of pedal release, was –232 ms on the right forelimb and –258 ms on the left forelimb, indicating that the muscles became active just before the onset of pedal release. In addition, we recorded the EMG activity of left forelimb in 6 healthy rats, in which the left muscles exhibited changes in activity only during left choice but not during right choice (change in EMG between pre-movement and post-movement: right pedal release, 4.4 ± 8.4%, left pedal release, 40.2 ± 14.7%), suggesting that the rats executed the pedal release using forelimb movement only on the required side (Fig. 2*B*).

To evaluate the effect of partial dopamine depletion in task performance, we compared the performance of the affected forelimb (left forelimb, contralateral to the lesion) with that of the spared forelimb (right forelimb, ipsilateral to the lesion) in the hemiparkinsonian rat. Population data revealed a preference for the right pedal movement (spared forelimb) in the initial 4 d of the training (*F* = 15.5, *p* = 9.90 × 10^−5^; day 1: *p* = 3.4 × 10^−8^; day 2: *p* = 1.9 × 10^−10^; day 3: *p* = 1.1 × 10^−8^; day 4: *p* = 2.5 × 10^−4^; two-way ANOVA, Tukey–Kramer *post hoc* test). However, at the end of the learning period; there were similar numbers of correct trails for both forelimbs (day 14: right, 505.6 ± 179; left, 519.1 ± 183; *p* = 0.8; Fig. 2*C*). Also, when the block changed, the rats learned to properly change their choice within an average of five trials, with no pedal bias (number of trials after block change to achieve a 50% correct response: 5 ± 1 trials; Fig. 2*D*).

Next, we compared the hemiparkinsonian and heathy rats’ performances, specifically right or left preference, total rate of correct response, and number of total trials. Two-way ANOVA analysis showed significant main effects of lesion (*F* = 58.62, *p* = 2 × 10^−13^), time (*F* = 3.96, *p* = 4.93 × 10^−6^), and interaction between time and lesion (*F* = 2.91, *p* = 4.8 × 10^−4^). *Post hoc* analyses revealed that in the early days of the learning period the lesioned rats presented a right movement preference (contralateral to the non-lesioned hemisphere; day 1: *p* = 7.4 × 10^−5^; day 2: *p* = 4.3 × 10^−7^; day 3: *p* = 3.3 × 10^−6^; day 4: *p* = 2.1 × 10^−4^; two-way ANOVA, Tukey–Kramer *post hoc* test). After two weeks this right-movement bias disappeared, and the lesioned rats’ performance became similar to those of the healthy rats (day 14: healthy, –2.0 ± 11.4%; lesioned, 8.1 ± 16.7%; *p* = 0.1; Fig. 2*E*). In the total correct rate, there was a significant main effect of lesion (*F* = 15.51, *p* = 9.9 × 10^−5^, two-way ANOVA) and time (*F* = 64.7, *p* = 3.2 × 10^−83^, two-way ANOVA). The total rate of correct responses in the lesioned rats was below that of the healthy rats in the middle of the learning period (day 7: *p* = 9 × 10^−4^; day 8: *p* = 1.1 × 10^−4^; day 9: *p* = 6.5 × 10^−5^; two-way ANOVA, Tukey–Kramer *post hoc* test), but became similar after two weeks (day 14: healthy, 78.4 ± 3.5; lesion, 78.4 ± 10.8%; *p* = 0.9; Fig. 2*F*). The total number of trials was similar in both groups all across the learning period (*F* = 0.03, *p* = 0.8, two-way ANOVA; Fig. 2*G*). These results show that the partial dopaminergic lesion used in this study produced a subtle motor imbalance that allowed the rats to correctly perform the task in a similar manner to the healthy animals, executing the pedal release using forelimb movement only on the required side; moreover, after two weeks of training, performance was similar in both forelimbs.

### Functional activity of M1 and M2 neurons

**Figure 3.**
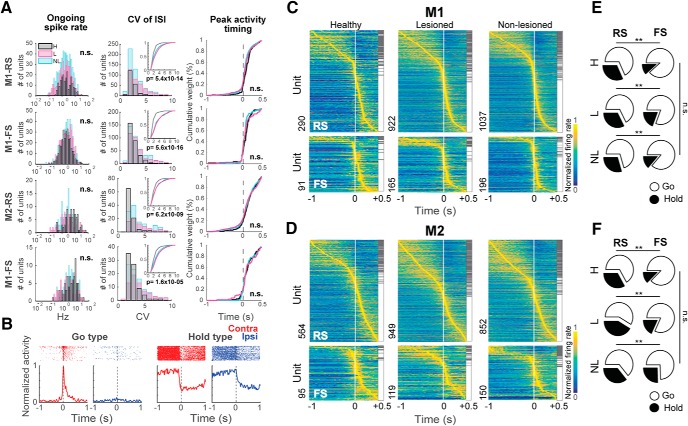
Different types of functional activity neurons in M1 and M2. ***A***, Spiking properties of RS and FS neurons of M1 and M2. Ongoing spike rate (left); CV of ISI, showing the cumulative distribution in the insets (middle); peak activity timing of preferred activity (right); n.s.: not significant, Kruskal–Wallis test (H, healthy; NL, non-lesioned hemisphere; L, lesioned hemisphere). ***B***, Examples of neurons with different functional activities recorded from the lesioned hemisphere, showing the raster plot and PETH around the onset of pedal release for a Go-type and a Hold-type neuron during contralateral (red) and ipsilateral (blue) movements. ***C***, ***D***, Task-related activity in RS (top) and FS (bottom) neurons in M1 and M2. Color map shows the normalized Gaussian-filtered PETH of the preferred response (σ = 12.5 ms, in 0.05-ms bins) for a single neuron (aligned with pedal release onset). Task-related neurons were sorted by peak activity time (early to late). Hold-type (gray) and Go-type (white) activities are indicated on the right side of each panel. ***E***, ***F***, Proportion of Hold-type (black) and Go-type (white) for RS and FS neurons; **p < 0.01, n.s.: not significant, χ^2^ test.

We recorded the extracellular multineuronal activity from the deep layers (putative layer 5) of M1 and M2 and compared the activity under three conditions: healthy rat (H), non-lesioned side in hemiparkinsonian (NL), and 6-OHDA-lesioned side in the hemiparkinsonian rat (L), while they performed alternated unilateral forelimb movements. We isolated a total of 7568 M1 neurons (M1-H, 834; M1-L, 3578; M1-NL, 3174) and 8231 M2 neurons (M2-H 1316; M2-L, 3771; M2-NL, 3144) during task performance. The neurons were classified as RS or FS neurons based on spike duration (Fig. 3*A*), which was consistent with previous reports (Isomura et al., 2009; Saiki et al., 2014, 2018; Soma et al., 2017). First, we examined some spiking properties of these neurons. We did not find any difference in the ongoing spike rate in M1 or M2 among the three conditions (M1-RS-H: 3.4 ± 3.8 Hz, M1-RS-L: 2.9 ± 3.5 Hz, M1-RS-NL: 3.8 ± 3.6 Hz, χ^2^ = 0.15, *p* = 0.2; M1-FS-H: 7.8 ± 10.3 Hz, M1-FS-L: 7.2 ± 10.6 Hz, M1-FS-NL: 4.8 ± 6.3 Hz, χ^2^ = 5.86, *p* = 0.053; M2-RS-H: 3.1 ± 3.9 Hz, M2-RS-L: 3.4 ± 3.3 Hz, M2-RS-NL: 3.5 ± 3.5 Hz, χ^2^ = 4.8, *p* = 0.1; M2-FS-H: 7.2 ± 6.7 Hz, M2-FS-L: 5.4 ± 6.3 Hz, M2-FS-NL: 6.2 ± 7.0 Hz, χ^2^ = 5.81, *p* = 0.054; Kruskal–Wallis test; Fig. 3*A*, left). Next, we compared the coefficient of variation (CV) of interspike interval (ISI) to evaluate the spiking regularity. The CV was higher in the non-lesioned and lesioned hemisphere in RS and FS neurons of M1 and M2, compared to the healthy condition (M1-RS: χ^2^ = 61.09, *p* = 5.4 × 10^−14^; M1-FS: χ^2^ = 70.2, *p* = 5.6 × 10^−16^; M2-RS: χ^2^ = 37.7, *p* = 6.2 × 10^−9^; M2-FS: χ^2^ = 22.08, *p* = 1.6 × 10^−5^; Kruskal–Wallis test, Dunn’s *post hoc* test; Fig. 3*A*, middle). Additionally, we compared the peak activity timing of the preferred activity. We did not find statistical difference in either population or condition (M1-RS: *p* = 0.54; M1-FS: *p* = 0.7; M2-RS: *p* = 0.2; M2-FS: *p* = 0.9; Kruskal–Wallis test; Fig. 3*A*, right). These results did not show the expected hypoactivity or delayed peak activity suggested by some hypothetical changes in parkinsonism. However, we found a higher irregularity in the spiking activity in the parkinsonian rats, in agreement to previous reports (Pasquereau et al., 2016).

We further analyzed the activity of M1 and M2 neurons during forelimb movements. First, we aligned the spike data with the onset of the right or left pedal release and defined the task-related neurons using the task relevance index. Next, we divided the task-related neurons as Go-type if they exhibited a phasic activity increase around movement, and Hold-type neurons if they exhibited a sustained activity before the pedal release (Fig. 3*B*; see Materials and Methods).

Figure 3*C*,*D* shows the preferred activity (lower task relevance index during contralateral or ipsilateral movements) sorted by peak position, differentiated by functional activity type. The RS neurons exhibited a higher proportion of Hold-type neurons relative to FS neurons in all instances (M1-H: χ^2^ = 13.52, *p* = 1 × 10^−3^; M2-H: χ^2^ = 12.94, *p* = 1 × 10^−3^; M1-NL: χ^2^ = 19.75, *p* = 5.14 × 10^−5^; M2-NL: χ^2^ = 9.66, *p* = 9 × 10^−3^; M1-L: χ^2^ = 14.40, *p* = 7.4 × 10^−4^; M2-L: χ^2^ = 20.08, *p* = 4.4 × 10^−5^; 2 × 2 χ^2^ test for Hold:Go ratio between RS and FS; Fig. 3*E*,*F*). The 6-OHDA injection did not modify the proportion of these neurons in either condition (M1-RS: χ^2^ = 2.58, *p* = 0.3; M1-FS: χ^2^ = 2.39, *p* = 0.3; M2-RS: χ^2^ = 3.97, *p* = 0.13; M2-FS: χ^2^ = 5.13, *p* = 0.1, χ^2^ test).

### Effect of the 6-OHDA lesion in the specificity representation of forelimb movements in M1 and M2

**Figure 4.**
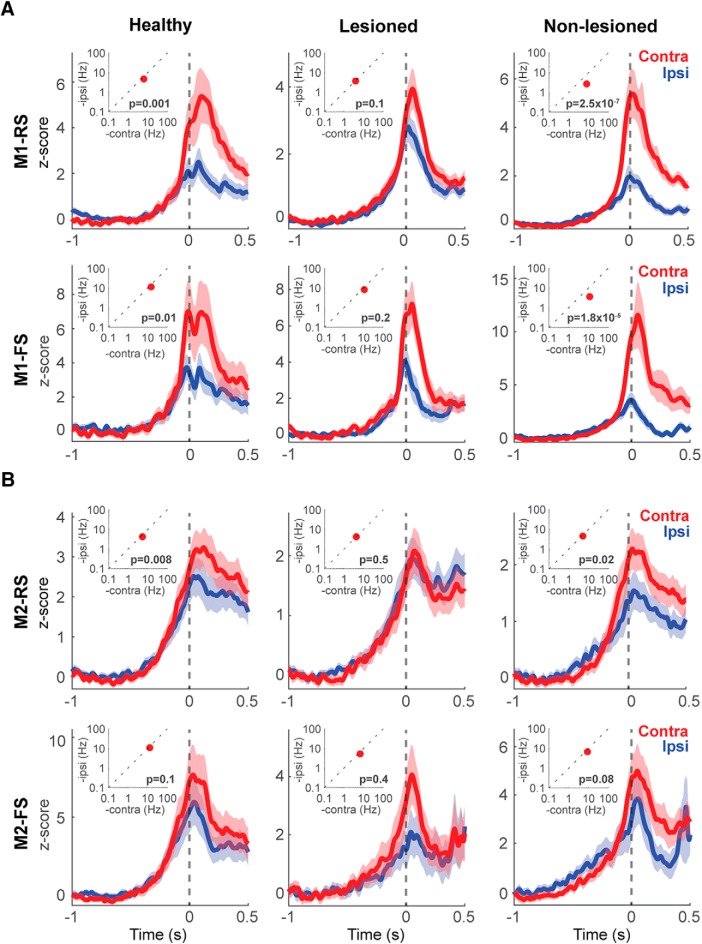
Forelimb selectivity of neuronal activity. Go-type neurons activity during contralateral and ipsilateral movements in RS and FS neurons in M1 (***A***) and M2 (***B***). The plots show the averaged PETHs of all Go-type activity during contralateral (red) and ipsilateral (blue) choice trials. Solid lines represent means, shaded areas represent ±SD. H, healthy; L, lesioned; NL, non-lesioned. The insets show the difference between the average peak values during contralateral and ipsilateral movements. Wilcoxon rank-sum test.

To analyze the selectivity of neuronal activity for the contralateral or ipsilateral forelimb movement, and evaluate the effect of the partial dopaminergic lesion, we first compared the pre-movement activity (for Hold-type neurons), or the peak activity around pedal movements (for Go-type neurons), between contralateral and ipsilateral movements. Figure 3*B* shows examples of functional activities obtained from contralateral (red) and ipsilateral (blue) movement trials. For Hold-type neurons, we compared the mean firing rate before contralateral and ipsilateral forelimb movements (–1 to 0 s, relatively to movement onset). We did not find any difference in the activity between contralateral and ipsilateral movements in any condition (data not shown). Therefore, for the study of the lateralized activity, we focused only on the phasic activity of the Go-type neurons (Fig. 4, insets). In the healthy condition there was a higher activity during contralateral movements in M1-RS (*z* = 3.26, *p* = 1 × 10^−3^), M1-FS (*z* = 2.36, *p* = 0.01) and M2-RS (*z* = 2.61, *p* = 8 × 10^−3^), but we did not find statistical difference in M2-FS (*z* = 1.35, p = 0.2; Wilcoxon rank-sum test). In the lesioned hemisphere, this higher peak activity during contralateral movements was lost in all instances, M1-RS (*z* = 1.62, *p* = 0.1), M1-FS (*z* = 1.26, *p* = 0.2), M2-RS (*z* = –0.63, *p* = 0.5), and M2-FS (*z* = 0.7, *p* = 0.4; Wilcoxon rank-sum test). On the other hand, in the non-lesioned hemisphere, the difference in the peak activity during contralateral and ipsilateral movements was higher in M1-RS (*z* = 5.15, *p* = 2.5 × 10^−7^) and M1-FS (*z* = 4.28, *p* = 1.8 × 10^−5^), but not in M2-RS (*z* = 2.25, *p* = 0.02) or M2-FS (*z* = 1.71, *p* = 0.08; Wilcoxon rank-sum test). These results show that the different functional type neurons, Hold-type and Go-type, also differed in the preferred activity during contralateral movements. In addition, the 6-OHDA injection modified this preference in the Go-type neurons, particularly in M1.

Moreover, to qualitatively depict the selectivity of each neuron in M1 and M2, we normalized and averaged their spike activities (Fig. 4). In the healthy condition, the PETHs exhibited a preferred activation during contralateral movements in RS and FS, in both M1 and M2 (Fig. 4, left column). On the lesioned side, the difference in activity between contralateral and ipsilateral movements was reduced (Fig. 4, middle column). In the non-lesioned side in the parkinsonian rats, the difference was strengthened, mainly in M1, where there was a notable preferred activation during contralateral movements (Fig. 4, right column). These results are in agreement with the peak activity changes produced in the parkinsonian state.

**Figure 5.**
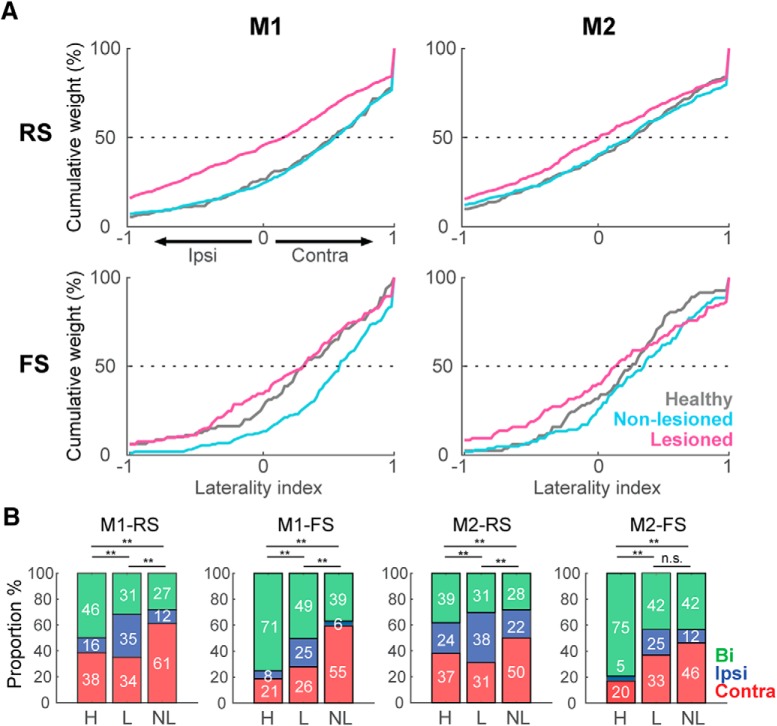
Laterality preference of Go-type activity in unidentified neurons in M1 and M2. ***A***, Cumulative distributions of laterality indices for RS and FS neurons in M1 and M2. A laterality index near +1 indicates preferred activity during contralateral movements, and an index near –1 indicates a preference for ipsilateral movements. Each line represents the lateralized activity of the indicated neuron population under healthy (gray), non-lesioned (cyan), and lesioned (magenta) conditions. ***B***, Proportion of neurons (in percentage) selective for contralateral (red), ipsilateral (blue), or bilateral movement (green); **p < 0.01, n.s.: not significant, χ^2^ test.

Further, to quantify the limb specificity, we used the laterality index based on the normalized phasic activity during contralateral or ipsilateral movement. Figure 5*A* shows the distribution of the laterality index in the healthy, non-lesioned, and lesioned conditions. A laterality index near +1 indicates preferred activity during contralateral movements, and an index near –1 indicates a preference for ipsilateral movements. In M1-RS neurons, the bias in the laterality index for a contralateral preference was similar in the healthy and non-lesioned conditions, whereas there was a leftward shift in the distribution in the lesioned hemisphere, showing a decrease in the contralateral preference (H: 0.38 ± 0.04, NL: 0.38 ± 0.02, L: 0.05 ± 0.02, *F* = 44.27, *p* = 2 × 10^−19^; H vs NL: *p* = 0.9; H vs L: *p* = 9.1 × 10^−9^; NL vs L: *p* = 9.6 × 10^−10^; one-way ANOVA, Tukey–Kramer *post hoc* test). This decrease in the contralateral bias in the lesioned hemisphere was also present in the M2-RS population, but the difference among the three conditions was minor relative to M1-RS (H: 0.16 ± 0.03, NL: 0.16 ± 0.03, L: 0.02 ± 0.03, *F* = 6.22, *p* = 2 × 10^−3^; H vs NL: *p* = 0.9; H vs L: *p* = 0.01; NL vs L: *p* = 5 × 10^−3^; one-way ANOVA, Tukey–Kramer *post hoc* test). By contrast, in the M1-FS neurons, the non-lesioned hemisphere exhibited the greatest contralateral preference (H: 0.28 ± 0.06, NL: 0.50 ± 0.03, L: 0.20 ± 0.05, *F* = 11.71, *p* = 1.2 × 10^−5^; H vs NL: *p* = 8 × 10^−3^; H vs L: *p* = 0.6; NL vs L: *p* = 9.3 × 10^−6^; one-way ANOVA, Tukey–Kramer *post hoc* test); whereas the M2-FS population exhibited no difference under either condition (H: 0.21 ± 0.05, NL: 0.29 ± 0.05, L: 0.12 ± 0.06, *F* = 2.33, *p* = 0.09, one-way ANOVA).

We also divided the RS and FS neurons into three categories according to the task relevance index (Fig. 5*B*, contralateral, red; ipsilateral, blue; bilateral, green). In accord with the laterality index, a larger proportion of neurons was selective for ipsilateral movements in the lesioned hemisphere in both M1 and M2 (M1-RS: χ^2^ = 167.1; *p* = 4.4 × 10^−35^, M1-FS: χ^2^ = 70.4; *p* = 1.9 × 10^−14^, M2-RS: χ^2^ = 64.68; *p* = 3 × 10^−13^, M2-FS: χ^2^ = 39.52; *p* = 5.4 × 10^−8^, χ^2^ test). These results demonstrate that the main effect of the partial 6-OHDA lesion was to decrease the contralateral preferred activation of both M1-RS and M2-RS, whereas M1-FS neurons in the non-lesioned hemisphere exhibited the opposite effect.

### Motor laterality in identified IT and PT neurons

**Figure 6.**
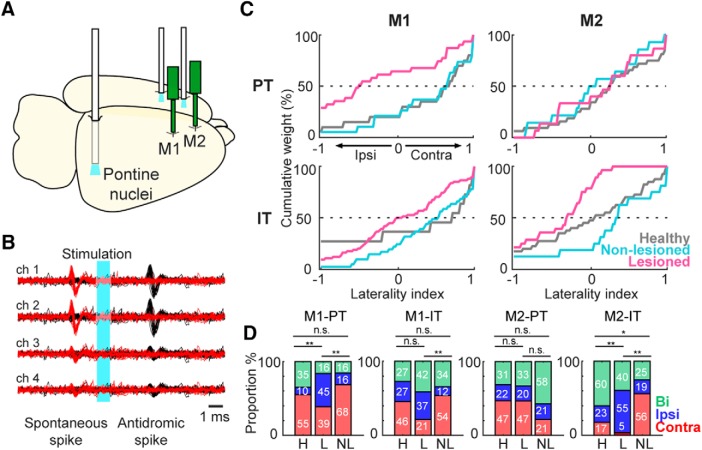
Identification and laterality preference of IT and PT neurons. ***A***, Schematic of the localization of recording probes and optical fiber for performance of Multi-Linc in M1 and M2 (contralateral cortex stimulation for the identification of IT neurons; ipsilateral pons stimulation for PT neurons). ***B***, Example identification of a PT neuron using the collision test. Black traces represent antidromic spikes after optical stimulation (cyan area). Red traces show the absence of the antidromic spike after optical stimulation triggered by a spontaneous spike, confirming a successful collision test. ***C***, Cumulative distributions of laterality indices for IT and PT neurons in M1 and M2. A laterality index near +1 indicates preferred activity during contralateral movements, and an index near –1 indicates a preference for ipsilateral movements. Each line indicates the lateralized activity of each neuron population under healthy (gray), non-lesioned (cyan), and lesioned (magenta) conditions. ***D***, Proportion of neurons (in percentage) selective for contralateral (red), ipsilateral (blue), or bilateral movement (green); *p < 0.05, **p < 0.01, n.s.: not significant, χ^2^ test.

Next, we investigated the limb specificity of the identified IT and PT projection neurons. To identify these types of projections neurons, we performed the collision test, using the Multi-Linc method (Soma et al., 2017; Saiki et al., 2018). We stimulated the contralateral motor cortex to identify IT neurons, and the ipsilateral pontine nuclei to identify PT neurons (Fig. 6*A*). Figure 6*B* shows the antidromically evoked spike and the successful collision test, in which the antidromic spike disappeared if the stimulation was triggered by the spontaneous spike. In M1 and M2, latency was shorter in PT neurons than in IT neurons (M1-PT-NL: 6.90 ± 3.80 ms, M1-IT-NL: 11.68 ± 5.40 ms, *z* = 7.57, *p* = 3.7 × 10^−14^; M1-PT-L: 7.62 ± 4.66 ms, M1-IT-L: 11.44 ± 4.99 ms, *z* = 5.79, *p* = 6.6 × 10^−9^; M2-PT-NL: 7.16 ± 3.74 ms, M2-IT-NL: 12.31 ± 5.31 ms, *z* = 9.12, *p* = 6.9 × 10^−20^; M2-PT-L: 7.10 ± 3.33 ms, M2-IT-L: 10.89 ± 4.78 ms, *z* = 6.41, *p* = 1.4 × 10^−10^; Wilcoxon rank-sum test). The antidromic spike latency histogram showed a unimodal distribution. Additionally, there was no difference in the latency of any population in the lesioned versus non-lesioned hemisphere, suggesting that the conduction velocity remained intact after the 6-OHDA lesion. There was no significant difference in the proportion of functional activity between IT and PT in M1 and M2, and dopamine depletion had no effect on this proportion (data not shown).

As with the RS and FS neurons, we evaluated the limb specificity of Go-type neurons using the normalized laterality indices (Fig. 6*C*). Similar to the unidentified RS and FS neurons, the lesioned state was associated with a reduction in contralateral preference. However, we observed a differential effect of the 6-OHDA lesion on the distribution of the laterality index, depending on the recorded area. In the case of M1-PT neurons, the non-lesioned hemisphere neurons kept the contralateral bias similar to the healthy condition, whereas on the lesioned side there was a significant decrease in the contralateral bias (H: 0.40 ± 0.14, NL: 0.30 ± 0.17, L: –0.28 ± 0.14, *F* = 6.32, *p* = 3 × 10^−3^; H vs NL: *p* = 0.9; H vs L: *p* = 6 × 10^−3^; NL vs L: *p* = 0.02; one-way ANOVA, Tukey–Kramer *post hoc* test). On the other hand, the M1-IT neurons did not exhibit significant difference in the laterality index under any of the three conditions (H: 0.24 ± 0.26, NL: 0.35 ± 0.10, L: 0.04 ± 0.07, *F* = 2.82, *p* = 0.1; one-way ANOVA; Fig. 6*C*, left column). In contrast to M1, the M2-PT neurons did not exhibit any difference in the laterality index (H: 0.25 ± 0.10, NL: –0.03 ± 0.19, L: 0.03 ± 0.19, *F* = 1.09, *p* = 0.34; one-way ANOVA), whereas in the M2-IT neurons there was a significant difference in the laterality index in the lesioned side neurons relative to the healthy and non-lesioned conditions (H: 0.01 ± 0.11, NL: 0.15 ± 0.20, L: –0.44 ± 0.09, *F* = 5.54, *p* = 5 × 10^−3^; H vs NL: *p* = 0.9; H vs L: *p* = 0.02; NL vs L: *p* = 0.01; one-way ANOVA, Tukey–Kramer *post hoc* test; Fig. 6*C*, right column). We also categorized the IT and PT neurons according to the task relevance index (Fig. 6*D*, contralateral, ipsilateral and bilateral). We found a smaller proportion of neurons selective for contralateral movements in the lesioned hemisphere in M1-PT, M1-IT, M2-IT, but there was no difference in this proportion in the M2-PT population (M1-PT: χ^2^ = 11.27, *p* = 0.02, M1-IT: χ^2^ = 15.28, *p* = 4 × 10^−3^, M2-PT: χ^2^ = 3.54, *p* = 0.47, M2-IT: χ^2^ = 24.91, *p* = 5.2 × 10^−5^, χ^2^ test). These changes in the laterality index found in the lesioned hemisphere revealed a differential effect of dopamine depletion in the RS population, depending on the motor area. The decrease in the PT contralateral preference was specific to M1, but this population was not affected in M2. On the other hand, IT neurons were the only population affected in M2.

## Discussion

In this study, we characterized how different types of neurons in the M1 and M2 areas represent remodeling of lateralized movement representation in the parkinsonian state. Specifically, we investigated the effect of partial dopamine depletion on the forelimb selectivity of the motor cortex; recorded the neuronal activity from the output layer of M1 and M2, differentiating between RS and FS neurons; and identified the IT and PT neurons using the Multi-Linc method. We compared neuronal activity in rats performing the right–left pedal task under three different conditions: healthy rats, non-lesioned hemisphere, and 6-OHDA-lesioned hemisphere in hemiparkinsonian rats. The main finding of this study is that the partial dopamine depletion decreased the characteristic contralateral lateralized activity of the motor cortex. Due to the strong contralateral preference of the M1-RS neurons in the normal rats, this effect was clearly observed in this population. The non-lesioned hemisphere had the opposite effect, increasing the contralateral preference, particularly in the M1-FS neurons, indicating a possible pathologic or compensatory change in the hemisphere contralateral to the lesion. In addition, we observed a contrasting effect of the 6-OHDA lesion effect in the PT and IT neuron activity of M1 and M2. In M1, only the PT neurons decreased their contralateral preference, whereas in M2, the affected population was the IT.

### Partial dopaminergic depletion and task performance

We produced a hemiparkinsonian rat model injecting 6-OHDA into the DLS. The rats exhibited rotational behavior after apomorphine injection, with at least 150 contralateral turns per hour. This rotational behavior has been used as a reliable method for demonstrating motor imbalance in PD rat models (Bankiewicz et al., 2004). Also, the loss of TH staining in motor cortex and striatum was evident around three weeks after 6-OHDA injection, where the TH staining was practically completely lost around the injection side, with a smaller, but significant decrease in M1. The loss of TH+ cells in the ipsilateral SNc was around 60%, similar to previous studies using partial lesions (Amalric and Koob, 1987; Lemaire et al., 2012). However, even with this important reduction in the TH+ cells, the rats did not exhibit overt parkinsonian symptoms. Compensatory mechanisms in the SNc (such as acceleration of DA turnover or receptor hypersensitivity), or broader changes in the cortico-BG network, may explain why clinical signs of parkinsonism emerge only after a massive degeneration of DA neurons (Melamed et al., 1982). In PD patients and PD monkey models, the loss of dopamine is predominantly observed in the posterior putamen, a region of the BG associated with the control of habitual behavior (Hornykiewicz, 1998; Kish et al., 1988; Brooks et al., 1990; Redgrave et al., 2010). Thus, partial dorsal striatum lesions may resemble this dopamine loss pattern. However, it is important to mention that this kind of lesion also affects the direct dopaminergic input to the motor cortex (Guo et al., 2015), which was evident in our study with the reduction of TH staining in M1. Therefore, the changes observed in motor cortex activity could be produced by a direct effect of the loss of dopamine in the cortex or by greater dysregulation of the cortico-BG circuit. Additionally, we observed an increase in the β-frequency power in the cortical LFP, and increased coherence between cortical regions, which is characteristic of the parkinsonian state (Silberstein et al., 2005; Mallet et al., 2008; Stoffers et al., 2008; Lemaire et al., 2012). Lesions in the dorsal striatum may resemble presymptomatic or mild stages of PD, in which early neuronal changes have already occurred, but the overt symptomatology may not still be present.

The study of PD in animal models is usually performed under a complete dopamine depletion in one or both hemispheres (Magill et al., 2001; Mallet et al., 2008; Emborg, 2017). This limits the assessment of movement-related activity due to the reluctance of the animals to move (Leblois et al., 2006). Likewise, under operant conditions, a more extensive striatal activity disruption may affect the velocity and number of movements (Panigrahi et al., 2015), or the motivational and motor processing (Hori et al., 2019). However, our partial DLS lesion permitted the rat to perform skillful forelimb movements after training, and allowed us to study brain activity during task performance under similar forelimb movement conditions. Consistent with our results, partial dopamine depletion does not impede learning of other tasks (Molina-Luna et al., 2009; Lemaire et al., 2012). In our pedal task, under healthy conditions, the rats exhibited a preference for either right or left movements in the early training period, which disappeared along with the training progress. In the lesioned rats, the preference for movement of the right forelimb (contralateral to the non-lesioned hemisphere) was consistent across all rats. However, the performance of the lesioned rats was similar in both forelimbs after two weeks of training, and similar to that of the healthy rats. This correction may be a consequence of the training itself, or instead the result of compensatory changes from the non-lesioned hemisphere (Remple et al., 2001). This partially lesioned hemiparkinsonian model under task performance may be useful in future studies of movement-related activity in mild parkinsonism.

### Decreased limb specificity in M1 and M2 in parkinsonian state

Selectivity during ipsilateral or contralateral movements suggests diversity in the degree of lateralization between the motor cortices, in which regions representing higher-order information exhibit less lateralization (PM, SMA in monkeys; M2 in rats) and have a greater role in bilateral coordination; by contrast, M1 encodes concrete motor information, and therefore requires higher specificity for unilateral forelimb movements (Kurata, 2007; Nakayama et al., 2015; Soma et al., 2017). In our study, the contralateral preference, mainly in M1, was decreased by dopamine depletion. These results are in agreement with fMRI studies showing higher ipsilateral cortical activation during voluntary movements in PD patients (Wu et al., 2015). The disruption of cortical interhemispheric inhibition is likely to be the primary mechanism responsible for promoting the decrease in motor cortex lateralization and the generation of mirror movements (Johnson et al., 1998; Almeida et al., 2002; Li et al., 2007; Wu et al., 2015). However, these changes are not restricted to the motor cortex. In the parkinsonian state, a large fraction of BG neurons was related to movements of more than one body part, including activity modulations related to movements of both the arm and the leg, movements of multiple joints, and movements of the ipsilateral limbs (Filion et al., 1988; Levy et al., 2001; Williams et al., 2005; Baker et al., 2010; Erez et al., 2011).

**Figure 7.**
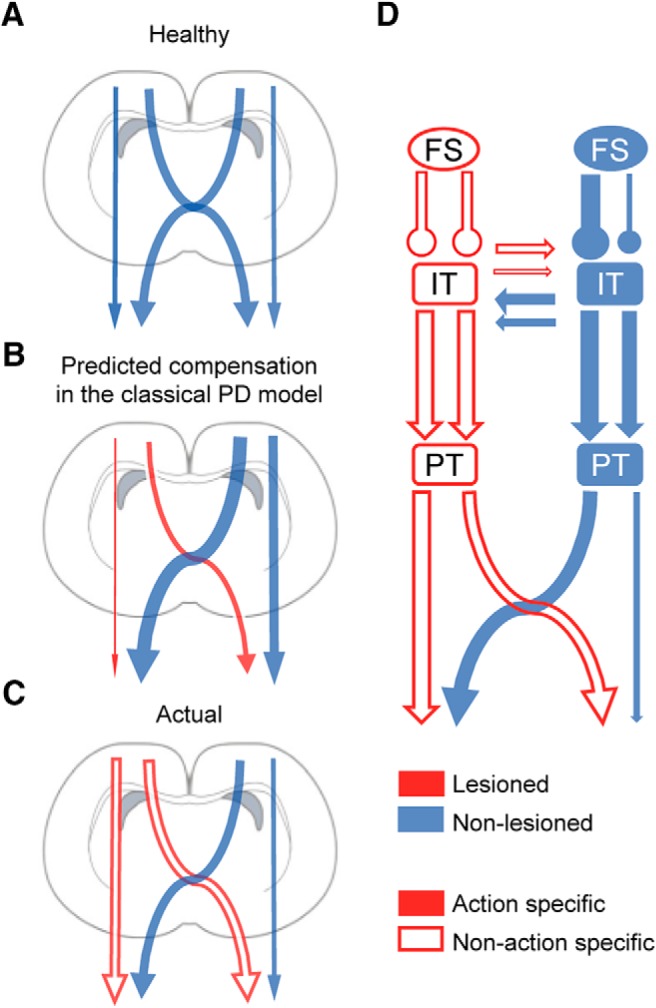
Disrupted lateralized activity of motor cortex. The present model represents the degree of ipsilateral or contralateral control depicted as the arrow thickness. ***A***, Normal contralateral lateralized activity of motor cortex. ***B***, Classic rate model of PD and hypothetical compensation from the contralateral hemisphere. ***C***, Our results indicate a decrease in the contralateral preferred control in the dopamine-depleted hemisphere. The empty arrows represent a reduced action selectivity (e.g., flexion or extension) in the parkinsonian state. ***D***, Projection neurons and putative interneurons on the lesioned side exhibited a reduction in contralateral preference which can coexist with a decrease in the action selectivity. The non-lesioned hemisphere exhibited an increased contralateral preference in FS neurons and a tendency toward an increase in the contralateral preference in projection neurons, acting as a possible compensatory mechanism, exerting a higher control over the abnormal activity of the dopamine depleted hemisphere.

According to the classic rate model of PD, dopamine depletion produces an imbalance between the direct and indirect pathways of the cortico-BG network, decreasing activity in the former and increasing activity in the latter (although we did not find an overall decreased activity). This imbalance explains reduced thalamic output to the motor cortex, culminating in the characteristic hypokinetic manifestation of PD (Albin et al., 1989; DeLong, 1990; Galvan and Wichmann, 2008; Pasquereau and Turner, 2011). The reduction in cortical motor output is associated with reduced control specificity, in which the signal to the contralateral part of the body is reduced relative to the ipsilateral signal. In the case of monkey or rodent hemiparkinsonian models or early-stage PD in humans, in which one hemisphere is more affected than the other (Kish et al., 1988), the activity in the less affected hemisphere is associated with increasing ipsilateral control (Bronfeld and Bar-Gad, 2011; Viaro et al., 2011; Wu et al., 2015). This reorganization of the cortical map may act as a compensatory mechanism to control the side of the body contralateral to the lesioned hemisphere (Fig. 7*B*; Netz et al., 1997). Nonetheless, these potentially compensatory changes in the non-lesioned side may produce other motor problems in the attempt to preserve the necessary output to both sides of the body, e.g., mirror movements (Vidal et al., 2003; Espay et al., 2005; Li et al., 2007).

In disagreement with the classic model of PD and the possible compensatory modifications, we observed opposite changes in the lesioned and non-lesioned hemispheres regarding preferred movement–related activity during contralateral and ipsilateral forelimb movements. In M1-RS and M2-RS neurons we observed a decrease in the selectivity for contralateral movements in the lesioned hemisphere and an increased proportion of ipsilateral-preferring neurons (Figs. 4, 5). On the other hand, we found a higher proportion of contralateral neurons and a stronger bias for contralateral movement representation on the non-lesioned side, relative to the healthy and lesioned conditions (Fig. 5*B*). Previous studies in patients with hemiparkinsonian symptoms revealed that during the movement of the forelimb ipsilateral to the less affected hemisphere, there is a stronger inhibitory influence from the most affected hemisphere to the opposite side (Wu et al., 2015). These results are in agreement with our study, which revealed reduced activity during ipsilateral movements in the non-lesioned hemisphere, resulting in a greater difference in activity between contralateral and ipsilateral movements. One mechanism that could produce these changes in the most affected hemisphere is a reduced activation of the striatum, which would decrease its influence, primarily in the cortical motor areas on the same side. Meanwhile, the increased activation of the striatum in the less affected hemisphere may promote higher selectivity for contralateral movements (Playford et al., 1992; Holden et al., 2006; Prodoehl et al., 2010). In addition, there is higher activation of the less affected hemisphere PM and of the more affected hemisphere cerebellum, which contributes to the weakened motor lateralization in the more affected hemisphere (Wu et al., 2015). The increased lateralized activity of FS neurons in the non-lesioned hemisphere may contribute to the potentially compensatory changes, not only modulating the activity on the same side, but also modifying the activity of IT neurons projecting to the contralateral hemisphere.

The activity of the lesioned hemisphere during contralateral movements remained similar to the activity in a healthy animal, implying that the decrease in contralateral selectivity was due to increased activity during ipsilateral movements. We speculate that to maintain an adequate performance, this abnormally increased drive to the ipsilateral forelimb from the lesioned hemisphere requires additional control from the non-lesioned side. Studies in monkeys indicate that a large number of nonprimary motor cortex neurons exhibit changes in activity that were not entirely related to muscle activity, but were associated with higher-order motor control (Tanji et al., 1987). Therefore, the preferred activation of the non-lesioned hemisphere during contralateral movements that we observed may not be entirely related to muscle activation but may act as a control of the abnormal ipsilateral activity from the lesioned-hemisphere (Fig. 7*C*).

### Differential effect of dopaminergic depletion on IT and PT neurons

We found that dopamine depletion produced a specific reduction in the contralateral preference only in PT neurons in M1, and only in IT neurons in M2. Considering the variation in anatomic traits and inputs, different cortical projection neurons may play different roles in the pathology and compensatory mechanisms in PD. Several studies support the idea of a differential effect of dopamine depletion depending on cortical region and neuron type. In resting MPTP-treated parkinsonian monkeys, only M1-PT neurons exhibit a reduction in firing rate and elevation in bursting activity, in agreement with the classic rate models, suggesting that these cells are particularly sensitive to the loss of dopamine (Pasquereau and Turner, 2011). Additionally, in subsequent studies, MPTP produced a reduction in kinematic encoding, principally in the PT neurons (Pasquereau et al., 2016). This PT sensitivity could be explained by direct input from the thalamic nuclei, which themselves receive direct inputs from the BG, and are affected by the propagation of abnormal BG activity (Strick and Sterling, 1974; Rathelot and Strick, 2009). However, other features such as different laminar distributions, morphologies, or firing patterns may contribute to the responses of these neurons to pathologic conditions (Stewart et al., 2000; Morishima and Kawaguchi, 2006; Groh et al., 2010). In this study, we observed a reduction in the contralateral preference in PT neurons of M1, but not in IT neurons, whereas in M2 the change in contralateral preference was observed only in the IT population. PT neurons are positioned to play a relatively direct role in the expression of PD motor signs due to their direct projections to the spinal cord (Magill et al., 2001). By contrast, IT neurons form the basis of interhemispheric interactions and provide important glutamatergic input to motor regions of the striatum, influencing the disorder physiology of the dopamine-depleted striatum (Mallet et al., 2006). The importance of dopamine in interhemispheric connectivity is strongly suggested by studies in hemiparkinsonian rats, which exhibit increased functional coupling between the left and right motor cortices, as well as between the motor cortex on the non-lesioned side and the STN on the lesioned side (Degos et al., 2009; Jávor-Duray et al., 2015). Therefore, the possible compensatory increased activity from the dopamine-dominant hemisphere, which is aimed at promoting movement in the lesioned hemisphere (through IT neurons), comes at the cost of reduced specificity of PT neuron activation. Mirror movements are the extreme manifestation of this dysregulation, which is commonly expressed as impaired bimanual coordination (Fig. 7*D*). The dopaminergic system has considerable and wide-spread modulatory cortical influences (Steiner and Kitai, 2001). Thus, the relationship between corticocortical interaction and clinical symptoms may be a direct effect of the impairment of dopaminergic tone in the cortical function, such as a reduction in the intracortical inhibition (Ridding et al., 1995; Cantello et al., 2002). Decrements in corticocortical abnormal coupling with STN stimulation or levodopa have shown to produce a considerable clinical improvement (Silberstein et al., 2005). The effect of dopaminergic therapy may relate, in part, to a complex effect of dopamine on the patterning of striatal output, or extra-striatal effects, such as direct dopaminergic effects on the cerebral cortex, acting to suppress the abnormal synchronization between cortical regions. PD subjects during off-medication also exhibited higher ipsilateral area recruitment of the cerebellum and primary motor cortex, being partially normalized by levodopa (Palmer et al., 2009). Altogether, the levodopa replacement therapy has shown to have positive effects on the impaired striatum activity, decreased transcallosal inhibition, and compensatory efforts from cortical motor regions, or other areas such as the cerebellum, which are possible reasons contributing to the decreased motor lateralization in PD. Therefore, it is possible that this kind of therapeutic strategies may reverse the changes observed in the lateralized activity of M1 and M2.

In this study, we evaluated the effect of a dopaminergic system lesion during voluntary movement, and found that the changes in neuronal activity characteristic of the parkinsonian state may be present without clear behavioral changes. Also, dopamine depletion had different effects depending on the motor area. Moreover, the reduction in dopamine in one hemisphere caused changes (either pathologic or compensatory) in the non-lesioned hemisphere. Further studies are needed to determine whether changes in the non-lesioned hemisphere act as a compensatory mechanism to maintain similar performance in both forelimbs, as well as how the training process modifies the normal course of the effect of reduced dopamine on the motor cortex activity.

## Synthesis

Reviewing Editor: Leonidas Stefanis, Biomedical Research Foundation of the Academy of Athens Decisions are customarily a result of the Reviewing Editor and the peer reviewers coming together and discussing their recommendations until a consensus is reached. When revisions are invited, a fact-based synthesis statement explaining their decision and outlining what is needed to prepare a revision will be listed below. The following reviewer(s) agreed to reveal their identity: Hossein Aleyasin, Atsushi Nambu.

Synthesis of review:

The authors have performed a series of especially complicated experiments to assess the function of the motor cortex in hemiparkinsonian rats. Their conclusions regarding the alterations in contralateral preference and in the activity of specific neuronal populations within M1 and M2 are novel, and potentially significant. Overall, the data are of high quality. There are however, a number of issues that need to be addressed:

1. The authors should clarify the timing during which the behavioral tests and the recordings were performed. Was it during training, or once they had reached the plateu?

2. It would be important to get a sense of how the alterations in cortical activity evolve over time, during the period of training, and to compare them with the cortical activity measured once equilibrium is reached at 2 weeks of time.

3. It is not clear if vehicle-injected rats were utilized. If not, specific mention of this should be made, and the rationale for not doing so explained.

4. Although it is mentioned in the methods that right sided injections were performed, it would be helpful to reiterate this throughout the text to avoid confusion. In fact, the authors use many terms, such as left/right, lesioned/non-lesioned, and ipsilateral/contralateral that end up being confusing. A better presentation of the results along these lines would be helpful.

5. Figure 1C seems to represent steps in injured rats. Is there an equivalent trace for control rats?

6. The authors could examine by TH immunostaining whether dopaminergic input to M1 and M2 is altered following the 6-OH-DA lesion.

7. In the Legends of Figure 5A, 6C, one cannot find the “colored arrows representing a significant shift”.

8. It is not clear whether hold-type activity, burst and oscillatory findings, task-related EMG, the timing of peak spike activity (Fig 3), and the kurtosis of average peri-event histograms (Fig 4) change after the 6-OH-DA lesion.

9. The authors could discuss whether L-Dopa treatment would be expected to reverse the changes seen in M1 and M2.

10. There is a question whether PT neurons can be classified into fast and slow, and, if so, if they show differential responses.

11. Figure 7 is easily misunderstood. The thick projections make an impression that the projections are increased, such as increased ipsilateral corticospinal projections, although the authors want to describe increased ipsilateral activity.
